# AI Model for Predicting Anti-PD1 Response in Melanoma Using Multi-Omics Biomarkers

**DOI:** 10.3390/cancers17050714

**Published:** 2025-02-20

**Authors:** Axel Gschwind, Stephan Ossowski

**Affiliations:** 1 Institute of Medical Genetics and Applied Genomics, University of Tübingen, 72076 Tübingen, Germany; axel.gschwind@med.uni-tuebingen.de; 2 Institute for Bioinformatics and Medical Informatics, University of Tübingen, 72076 Tübingen, Germany

**Keywords:** artificial intelligence, immune checkpoint inhibitors, anti-PD1, melanoma, genomics, transcriptomics, multi-omics, least absolute shrinkage and selection operator (LASSO), SHAP values, explainable machine learning

## Abstract

Background: Immune checkpoint inhibitors (ICIs) have demonstrated significantly improved clinical efficacy in a minority of patients with advanced melanoma, whereas non-responders potentially suffer from severe side effects and delays in other treatment options. Predicting the response to anti-PD1 treatment in melanoma remains a challenge because the current FDA-approved gold standard, the nonsynonymous tumor mutation burden (nsTMB), offers limited accuracy. Methods: In this study, we developed a multi-omics-based machine learning model that integrates genomic and transcriptomic biomarkers to predict the response to anti-PD1 treatment in patients with advanced melanoma. We employed least absolute shrinkage and selection operator (LASSO) regression with 49 biomarkers extracted from tumor–normal whole-exome and RNA sequencing as input features. The performance of the multi-omics AI model was thoroughly compared to that of nsTMB alone and to models that use only genomic or transcriptomic biomarkers. Results: We used publicly available DNA and RNA-seq datasets of melanoma patients for the training and validation of our model, forming a meta-cohort of 449 patients for which the outcome was recorded as a RECIST score. The model substantially improved the prediction of anti-PD1 outcomes compared to nsTMB alone, with an ROC AUC of 0.7 in the training set and an ROC AUC of 0.64 in the test set. Using SHAP values, we demonstrated the explainability of the model’s predictions on a per-sample basis. Conclusions: We demonstrated that models using only RNA-seq or multi-omics biomarkers outperformed nsTMB in predicting the response of melanoma patients to ICI. Furthermore, our machine learning approach improves clinical usability by providing explanations of its predictions on a per-patient basis. Our findings underscore the utility of multi-omics data for selecting patients for treatment with anti-PD1 drugs. However, to train clinical-grade AI models for routine applications, prospective studies collecting larger melanoma cohorts with consistent application of exome and RNA sequencing are required.

## Simple Summary

Many patients with skin cancer are treated with immune checkpoint inhibitors. The treatment can trigger an immune response that attacks cancer cells. This is achieved by inhibiting immune checkpoints, which act as an “off-switch” of the immune system. To date, medical doctors have selected patients with a high number of DNA mutations for this kind of therapy, a biomarker called tumor mutation burden (TMB). Still, only a minority of patients benefit from the treatment, even if they are selected based on high TMB. In our study, we first collected a large cohort of patients with melanoma treated with immune checkpoint inhibitors. We then derived additional tumor biomarkers from the sequencing data of the patients. We combined these new biomarkers into an artificial intelligence model to better predict whether a patient will respond to the therapy. Our results showed that combining multiple biomarkers can improve the treatment selection for patients with melanoma by providing more accurate outcome predictions.

## 1. Introduction

Immune checkpoint inhibitors (ICIs) have revolutionized the therapy of solid cancers [1]. ICIs targeting the immune checkpoints cytotoxic T-lymphocyte associated protein (*CTLA4*) or programmed cell death protein 1/ligand 1 (*PD1*/*PDL1*) have become an integral part of modern cancer treatment. Anti-PD1 drugs like pembrolizumab or nivolumab have demonstrated clinical efficacy, leading to a substantial clinical benefit for a subset of patients with advanced melanoma [2,3].

Despite these efforts, the majority of patients selected for ICI treatment do not show any benefit from the therapy [4]. There is only one FDA-approved biomarker for the selection of patients for ICI in melanoma: the nonsynonymous tumor mutation burden (nsTMB). A higher nsTMB is associated with better anti-PD1 response rates, with a commonly applied threshold of 10 mutations per megabase [5]. However, nsTMB alone is insufficient to predict therapeutic outcomes accurately. For instance, objective responses to pembrolizumab were observed in only 29% of patients with high nsTMB compared to 6% of patients with low nsTMB in a recent study [6]. Moreover, patients undergoing anti-PD1 treatment could suffer from potentially life-threatening adverse events, highlighting the urgent need for more reliable prediction methods [7]. Many other non-approved genomic and transcriptomic biomarkers have been shown to correlate with ICI outcomes in various cancer types, suggesting a potential avenue for improved outcome prediction [8]. Most of these biomarkers can be derived from next-generation sequencing (NGS) data, particularly whole-exome sequencing (WES) and bulk RNA sequencing (RNA-seq) [9,10].

Given the complex tumor–immune system interactions, we hypothesized that a multi-omics approach might be a more accurate predictor of anti-PD1 response than single biomarkers. Thus, we developed a comprehensive approach that integrates both genomic and transcriptomic biomarkers as features of a multi-omics-based machine learning model.

Several recent studies have developed machine learning (ML) models to enhance the prediction of ICI outcomes. Most models relied on non-NGS features, such as routine clinical biomarkers, serum parameters, or histopathology, to predict ICI outcomes [11,12,13]. Other studies focused on integrating either genomic or transcriptomic biomarkers [14,15,16]. Lichtfield et al. [17] developed a multi-omics ML model trained with data from multiple cancer types (“pan-cancer analysis”), using various DNA- and RNA-derived biomarkers. To our knowledge, no multi-omics models specializing in specific cancer types are available yet. The aforementioned models leveraged various ML algorithms, such as random forests, multivariate logistic regression, XGBoost, or neural networks.

Although these developments have improved ICI outcome prediction, most models provide limited clinical explainability of their predictions. To address these challenges, we developed a least absolute shrinkage and selection operator (LASSO) regression model [18] combined with SHAP analysis to enhance the accuracy of anti-PD1 outcome predictions in melanoma, to improve feature selection, and to provide explainable results for specific patients. We utilized WES to generate genomic biomarkers from paired tumor and normal tissues, and RNA-seq to generate transcriptomic biomarkers of the tumor tissue. In total, our LASSO approach initially incorporated 49 multi-omics biomarkers, covering various aspects of tumor biology, including the well-established nsTMB, neoantigens, resistance mechanisms, tumor immune microenvironment (immune cell infiltration), germline factors, and additional relevant metrics.

We obtained publicly available datasets of anti-PD1-treated melanoma patients to assemble a meta-cohort combining WES and RNA-seq, which we used to train and test the machine learning model. We classified patients as therapy responders or non-responders according to the well-established Response Evaluation Criteria In Solid Tumors (RECIST) [19]. This binary classification facilitated the comparison of therapeutic outcomes between different studies.

Finally, we thoroughly evaluated our model using several performance metrics to compare our LASSO approach with the current gold standard nsTMB. Our findings showed that the integrated multi-omics approach substantially improved ICI prediction by combining several biomarkers. Notably, a model based on transcriptomic (RNA-seq) biomarkers alone performed at least as good as or better than the genome-based nsTMB. A complementary analysis proved the general explainability of the AI predictions in a clinical context.

## 2. Materials and Methods

### 2.1. Patient Collective

We compiled a cohort from several previously published independent studies that investigated the response to anti-PD1 treatment in patients with metastatic melanoma of different stages. Raw sequencing data (paired tumor–normal WES and RNA-seq) were obtained from the Sequence Read Archive (SRA) and from the database of Genotypes and Phenotypes (dbGaP) after approval by the corresponding data access committees [20,21]. We included samples in our meta-cohort both from formalin-fixed paraffin-embedded (FFPE) and fresh frozen tissue. Normal samples are usually derived from peripheral blood. We discarded tumor-only DNA sequencing data and only used samples from pre-treatment biopsies. Patients were classified as responders or non-responders according to the RECIST criteria. Patients with progressive disease, stable disease, or mixed responses were classified as non-responders, whereas those with partial or complete responses were classified as responders.

**Figure 1:**
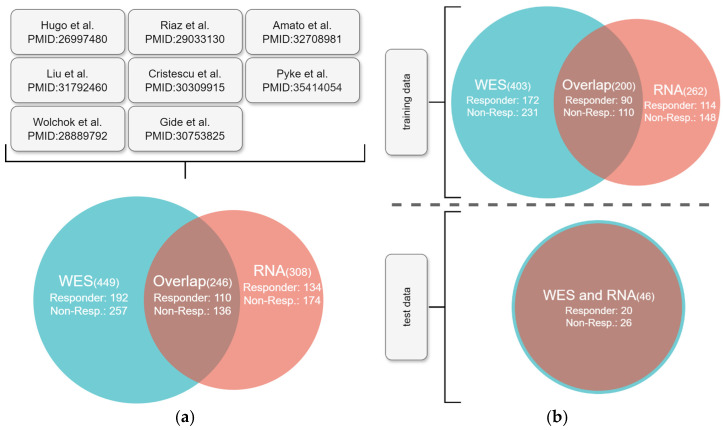
Overview of the overall cohort after quality control. The numbers in brackets denote the corresponding sample counts. (**a**) We integrated sequencing data from eight studies. We denote each study by the name of the first author of its publication [22,23,24,25,26,27,28,29]. RNA-seq data were not available for every WES, and vice versa; however, we achieved an overlap of 246 samples. (**b**) The cohort was randomly split into training and test datasets.

After quality control, the cohort reached a total size of 449 WES patients, of which 192 were responders and 257 were non-responders. For the RNA-seq samples, we obtained a cohort size of 308, of which 134 were responders and 174 were non-responders. Intersecting WES and RNA-seq data were available for 246 patients, of whom 110 were responders and 136 were non-responders. Figure 1a provides a detailed overview of the cohort. Each sub-cohort is denoted by the name of the first author of its seminal publication: Amato, Cristescu, Gide, Hugo, Liu, Pyke, Riaz, and Wolchok [22,23,24,25,26,27,28,29].

AI models require a test dataset that contains data unseen by the model during training. Thus, we randomly split the data into training and test datasets, resulting in 46 samples in the test dataset. We randomly selected test samples for which both WES and RNA-seq data were available. The split was stratified according to overall response and nsTMB. The final training–test split is illustrated in Figure 1b.

### 2.2. Machine Learning Model

Here, we used LASSO regression to predict response to anti-PD1 therapy. LASSO performs both regularization and feature selection by setting unimportant features to exactly zero. An important criterion for choosing LASSO as our ML model is its ability to handle multicollinearity in the input features, which is crucial for biological data and specifically for multi-omics data with various dependencies [18]. In contrast, tree-based models, such as XGBoost or RFs, can struggle with highly correlated input features and require extensive hyperparameter tuning. Furthermore, feature selection and interpretability as well as clinical explainability of the predictions were important criteria in this study. While methods such as XGBoost and RF do not eliminate coefficients of unimportant features [30] and SVMs do not provide feature selection at all, LASSO automatically selects a subset of the most predictive features, thereby improving interpretability. In combination with SHAP analysis (see below), LASSO therefore provided the best clinical explainability of results.

**Figure 2:**
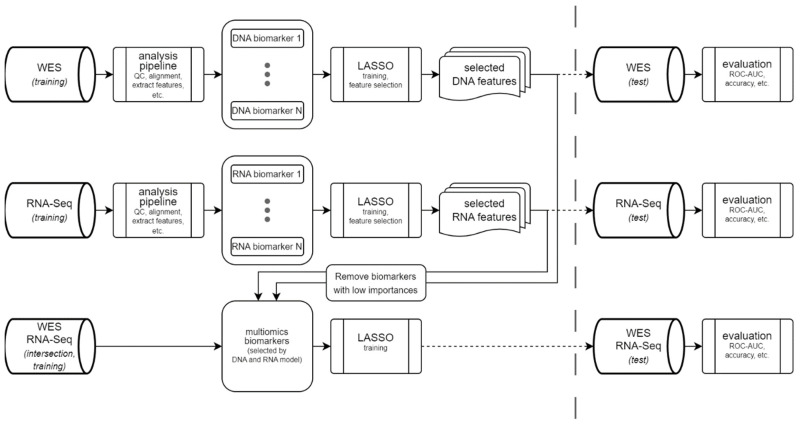
Flowchart of the AI models. First, we trained both the LASSO model using DNA data and the LASSO model using RNA data. The biomarkers selected that way were then used to train a multi-omics model using the overlap between all training samples for which both WES and RNA-seq data were available. We only used biomarkers with a permutation feature importance of >0.0055 for the multi-omics model, resulting in 10 features. All three models were then evaluated in the test set using different performance measures, such as ROC AUC.

Figure 2 shows an outline of our model design. To exploit the entire training dataset, we created one LASSO model exclusively using DNA biomarkers and another using only RNA-seq features. The contribution of each biomarker was estimated using permutation feature importance [31]. Every feature from the DNA and the RNA model with non-zero coefficients and a feature importance of >0.0055 was then used as an input feature for a multi-omics LASSO model, leading to a total of 10 features for the multi-omics model. This model combined DNA and RNA features and was trained on the overlap of samples for which both DNA and RNA-seq data were available.

We used 40 biomarkers as features of the DNA model. The following WES-derived metrics, describing both somatic and germline properties as well as resistance mechanisms, were used:

- Somatic features: *BRAF*:V600E status, tumor purity, nsTMB, insertion and deletion (indel) burden, frameshift indel (fsIndel) burden, in-frame indel burden, splice burden, missense burden, synonymous burden, multiple amino acid burden, frameshift mutation proportion, nonsynonymous to synonymous substitution ratio (dN/dS ratio), copy number variant (CNV) burden, deletion burden, neoantigen burden, maximum neoantigen-binding affinity, mean differential agretopicity index (DAI), median DAI, maximum DAI, upper decile DAI, maximum recognition potential, maximum HEX alignment score, and maximum dissimilarity score.
- Germline features: mean HLA evolutionary divergence (HED), HLA-B27 supertype, HLA-B44 supertype, HLA-B62 supertype, homozygous HLA-B, and homozygous HLA-C.
- Mechanisms of ICI resistance (enriched in non-responders): alterations in *B2M*, alterations in *TP53*, alterations in *STK11*, alterations in *PTEN*, alterations in *KRAS*, alterations in *MDM2*, alterations in *MDM4*, and alterations in *EGFR*.
- Mechanisms of ICI response (enriched in responders) were sparse in the training dataset and merged into one biomarker: alterations in *JAK1*/*JAK2,* deletions at chr6p21.3 (this locus contains HLA class I-related genes), and alterations in *CTNNB1*.

The RNA-based ML model was trained using nine input features. We included lymphocyte infiltration, T cell receptor (TCR) α chain entropy, TCR β chain entropy, immunoglobulin heavy chain (IGHG) entropy, interferon-γ to immunosuppression (IFNG-IMS) ratio, in-frame fusion neoepitope count, gene expression profile (GEP) of HLA class I genes, GEP of *PDL1*, and GEP of *B2M*. The implementation details and a thorough discussion of every biomarker used as an input feature in both the DNA and RNA model are provided in Appendix A.

We used LASSO as implemented in LassoCV in the Python library scikit-learn 1.3.2 [32]. We performed z-transformation with StandardScaler and trained each model using five-fold cross-validation. Receiver operating characteristic (ROC) area under the curve (AUC) values were used as the main measures of the predictive power of the DNA, RNA, and multi-omics models. To further analyze model performance, we stratified the predicted probabilities into predicted responders and non-responders. We determined the threshold for these binary predictions by maximizing Youden’s index for the training dataset, thereby ensuring an optimal trade-off between specificity and sensitivity [33]. Using this cutoff, we classified the model predictions as true positives (TP), true negatives (TN), false positives (FP), and false negatives (FN).

Machine learning models often suffer from a lack of explainability, which makes it difficult to determine the features that influence a specific prediction. Although we calculated permutation feature importance scores for our models, they are not meaningful on a per-sample basis, which is, however, crucial in clinical decision-making. Shapley Additive Explanations (SHAP) is an approach that addresses this issue and provides feature contributions for predictions on a per-sample basis [34]. Therefore, we applied SHAP to evaluate the explainability of the three models and thereby their clinical applicability. This helps clinicians comprehend the model’s prediction based on the biomarkers on which the model relied the most for a given case.

### 2.3. Bioinformatics Pipeline

#### Basic Analysis Pipeline

We used the megSAP pipeline for the basic analysis of both WES and RNA-seq data. megSAP (commit 2023_04-8-g6e28b110) is publicly available on GitHub https://github.com/imgag/megSAP (accessed on 2 May 2023). Briefly, read alignment against GRCh38 was performed using BWA mem2 for the WES samples. Subsequently, Manta 1.6.0 and Strelka 2.9.10 were used to call somatic variants (structural variants (SVs), single nucleotide variants (SNVs), and small indels) [35]. CNVs were called using ClinCNV 1.18.3 [36]. All variants were annotated using VEP v109 and the COSMIC Cancer Mutation Census (CMC) v99 [37,38]. For the RNA-seq samples, the reads were aligned against GRCh38 using STAR 2.7.10b. The read counts were then quantified using subread featureCounts 2.0.4 and normalized to transcripts per million (TPM) by megSAP [39]. In-frame and frameshift gene fusions were identified using Arriba 2.4.0. HLA genotyping was performed on the germline WES and RNA-seq samples using our in-house Python3 implementation of hla-genotyper [40]. The results were visually inspected using the clinical decision support system GSvar (commit 2023_03-23-g5390f8b), available on GitHub https://github.com/imgag/ngs-bits (accessed on 18 April 2023).

### 2.4. Target Regions

We constructed intersecting target regions for all studies included in our cohort. In short, we determined the region covered by at least 20× for every normal sample. Based on this information, we included a base in the target region if it was covered in at least half of the samples. The final target region was then constructed by intersecting it with the exonic coordinates ±2 bases obtained from ensemble v109. These target regions were used to calculate the coverage files for the CNV calling by ClinCNV and for quality control. For any other biomarker that required a target region (e.g., TMB calculation), we used the intersection between the target regions of all sub-cohorts to avoid batch effects.

### 2.5. Quality Control

For paired tumor–normal WES, we discarded all samples that did not meet the minimum coverage requirements of 60× for the tumor sample and 20× for the normal sample. Samples were also excluded if known SNPs found in the tumor and the normal samples did not show a correlation of at least 0.8, indicating that both samples might have originated from different individuals, that is, sample confusion. Multiple sequenced samples from the same tumor were available for a few patients in some studies. In these cases, we excluded all samples except those with the highest SRA number.

On the level of somatic variants (SNVs and small indels), we excluded all variants that did not fulfill the following quality criteria: (i) depth in tumor and normal sample > 20×, (ii) tumor variant allele frequency (VAF) ≥ 5%, (iii) normal VAF < 0.17 × tumor VAF, and (iv) the variant was detected in at least three or more reads. Copy number variants were discarded if ClinCNV reported a log likelihood of <100.

Gene expression data derived from RNA-seq generated at different sequencing facilities are prone to batch effects. Hence, we conducted a principal component analysis (PCA) of gene expression (in TPM) for all genes and all samples. The results revealed batch effects between samples of different sub-cohorts, as indicated by sub-cohort-based clustering (Figure S1, left). Subsequently, we corrected these batch effects using pyComBat [41]. PCA after batch correction did not show any obvious batch effects (Figure S1, right).

## 3. Results

### 3.1. AI Model for Prediction of Anti-PD1 Response

We trained a machine learning model predicting therapy response using a meta-cohort of 449 cases from eight studies, for which response to anti-PD1 was available as a RECIST classification. Our cohort consists of a total of 449 WES samples and 308 RNA-seq samples. However, not every WES sample has a paired RNA-seq sample and vice versa. There are 203 cases with only whole-exome sequencing (WES), 62 cases with only RNA-seq data, and 246 cases with both data types available. Considering this heterogeneity in the OMICs data availability, we trained three models: (1) a DNA biomarker model, (2) an RNA biomarker model, and (3) a multi-omics model. To use the same sub-cohort to evaluate all three models, we only chose cases for the test set that had both WES and RNA-seq data available. This test set consisted of 20% of otherwise randomly selected cases and was not used for model training. An overview of the cohort and the split of the cohort into training and test sets is shown in Figure 1.

**Figure 3:**
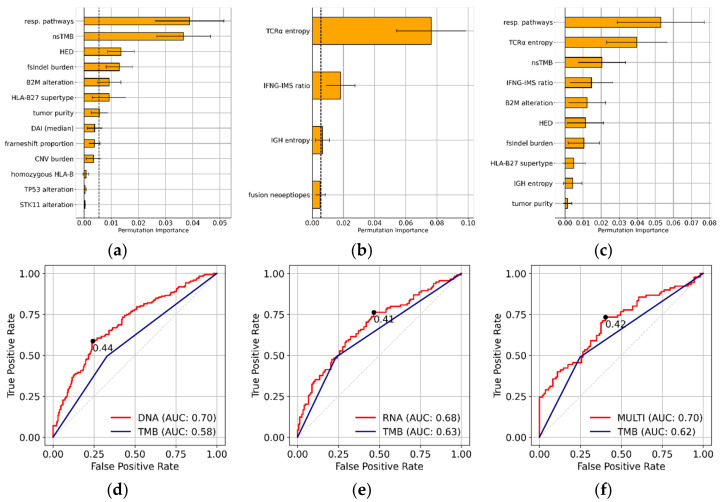
Feature permutation importance scores of the DNA model (**a**), the RNA model (**b**), and the multi-omics model (**c**). The dotted lines at 0.0055 indicate the cutoff used to include a feature in the multi-omics model. The error bars represent the standard deviation for each biomarker. (**d**–**f**) ROC curves for all three models. The red line represents the ROC curve for the LASSO model. The blue line represents the ROC curve for nsTMB using the FDA-approved threshold of 10 mutations per Mb. The black dots denote the optimal probability cutoffs.

An overview of the training and testing procedures is shown in Figure 2. Briefly, 49 DNA- or RNA-based biomarkers (see the Materials and Methods section for a full list) were extracted from short-read NGS data using the NGS analysis platform megSAP (see the Materials and Methods section for details). Next, we trained the DNA model using all cases of the training set with available WES data and the RNA model using all cases of the training set with available RNA data using LASSO. For both models, we performed feature selection (Figure 3a,b) and trained a LASSO model for cases with both WES and RNA-seq data using only features with a feature importance of more than 0.0055 in one of the two single-data models (Figure 3c; see Section 2 for details on model training). Finally, we compared the performance of the three models on the training and the test set and compared them with the well-established nsTMB.

### 3.2. Feature Selection and Feature Importance

First, we trained the DNA and RNA LASSO models on their corresponding biomarkers and training set using five-fold cross-validation. We obtained moderate LASSO regularization parameters of 0.021 for the DNA model and 0.024 for the RNA model. These parameters indicate that both models discarded some less important features but retained important features. As expected, the coefficients (impact on the model) of several biomarkers shrunk to zero, such as the *BRAF* mutation status for the DNA model or mean HLA expression for the RNA model. Hence, we retained 13 features with non-zero coefficients for the DNA model and four non-zero coefficients for the RNA model. We then evaluated the feature permutation importance scores of the non-zero LASSO coefficients (Figure 3a,b). Every biomarker above the permutation feature importance threshold of 0.0055 was used as an input feature for the multi-omics model. This further reduced the feature count of the multi-omics model to seven DNA and three RNA features. The final multi-omics model incorporated the following 10 initial genomic and transcriptomic biomarkers: response pathway, nsTMB, HED, fsIndel burden, defects in *B2M*, HLA-B27 supertype, tumor purity, TCR α chain entropy, IFNG-IMS ratio, and IGH entropy. The multi-omic model had a small LASSO regularization parameter of 0.002 after five-fold cross-validation, indicating that most of the features were retained. Indeed, the multi-omics model selected all the initial input features and resulted in ten non-zero (DNA and RNA) coefficients (Figure 3c).

### 3.3. Model Performance on Training and Test Set

**Figure 4:**
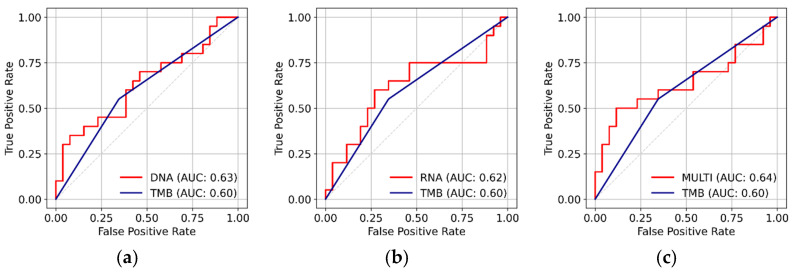
Model performance in the test set. (**a**–**c**) ROC curves of the DNA model (**a**), the RNA model (**b**), and the multi-omics model (**c**). None of the models exhibited overfitting. The ROC AUC of the LASSO models was higher than the ROC AUC of the nsTMB alone.

Next, we calculated the ROC curves using the response probabilities of the models for the training set, with the actual response data (responders and non-responders) serving as the ground truth. All three models outperformed the gold-standard nsTMB in terms of ROC AUC (Figure 3d–f). The DNA model achieved an ROC AUC of 0.70 versus 0.58 (nsTMB), the RNA model an ROC AUC of 0.68 versus 0.63 (nsTMB), and the multi-omics model an ROC AUC of 0.70 versus 0.62 (nsTMB) on the training dataset. The ROC AUC for the nsTMB of the RNA model was calculated using only the subset of samples for which paired WES data were available. The distribution of the prediction probabilities varied across the three models (Figure S2), emphasizing the necessity of model-specific probability cutoff values. By maximizing Youden’s index, we identified optimal probability thresholds of 0.44 for the DNA model, 0.41 for the RNA model, and 0.42 for the multi-omics model. Subsequently, these thresholds were used to classify the model predictions as TP, TN, FP, and FN. Next, we evaluated the DNA, RNA, and multi-omics models using the independent test set. Figure 4a–c show the performance of the models in the form of ROC curves. All three models outperformed nsTMB alone in terms of ROC AUCs: 0.63 versus 0.60 for the DNA model, 0.62 versus 0.60 for the RNA model, and 0.64 versus 0.60 for the multi-omics model. These results imply that the model training did not overfit.

### 3.4. SHAP Values

**Figure 5:**
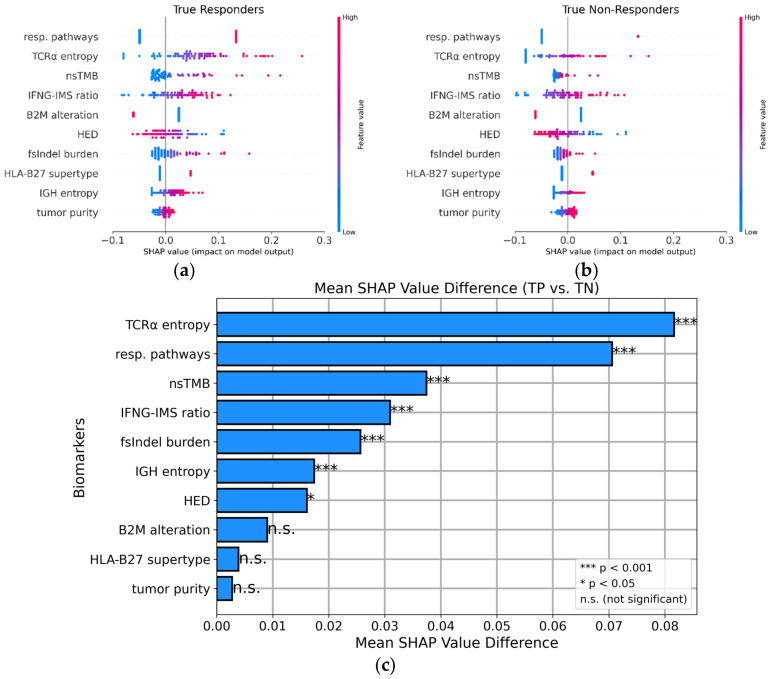
Summary plots (“bee swarm”) of the SHAP values for the multi-omics model using the overall cohort. True responders (**a**) were compared with true non-responders (**b**). Each dot represents the contribution of a feature to the prediction for a single sample. The dot color denotes the original feature value: blue, a low value, and red, a high value. (**c**) Differences in mean SHAP values between TP and TN. The asterisks (*) denote the significance level after a two-sided Mann–Whitney U test.

Subsequently, we applied the SHAP methodology to the complete cohort analyzed using the multi-omics model to identify typical biomarkers contributing to the prediction of TP (correctly identified responders) and TN (correctly identified non-responders). SHAP values estimate the impact of each feature on the prediction on a per-sample basis. Figure 5a,b show summary plots (“bee swarm”) of the SHAP values for the multi-omics model for true responders and true non-responders. Each dot represents the contribution of a biomarker to a specific model prediction. Positive SHAP values contribute to the prediction of responders, whereas negative SHAP values indicate a negative contribution to the prediction of non-responders. The dot color denotes the original input value of each feature; high input values are plotted in red, and low input values are plotted in blue. This allows us to determine the direction in which the features influence a prediction. For some of the biomarkers, we observed clear differences between the two groups. For instance, true non-responders had very low or zero TCR α chain entropy values that contributed to the prediction of TN. On the other hand, high TCR α chain entropy values contributed to the prediction of TP. To quantify such discriminative biomarkers between TP and TN, we calculated the differences in mean SHAP value for each feature (Figure 5c). We tested the significance of the differences using a two-sided Mann–Whitney U test with Bonferroni correction, resulting in seven statistically significant discriminative biomarkers between true responders and non-responders: TCR α chain entropy, response pathways, nsTMB, IFNG-IMG ratio, fsIndel burden, IGH entropy, and HED.

**Figure 6:**
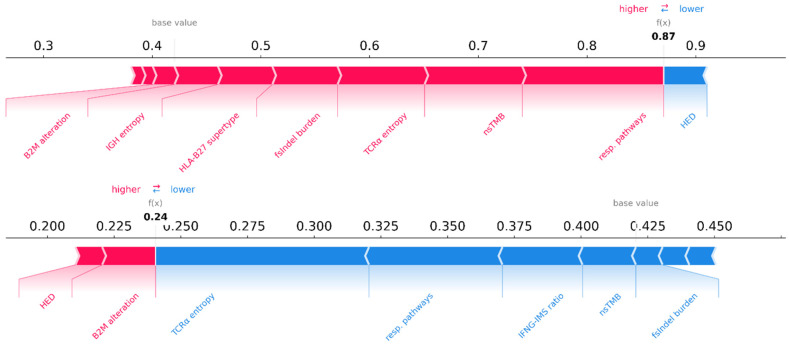
Two representative SHAP force plots of a TP (**top**) and a TN (**bottom**). Using these plots, we can evaluate and visualize the contribution of each feature on a specific sample directly. Both genomic and transcriptomic biomarkers contributed to the correctly identified responder and non-responder.

Figure 6 shows representative plots for two patients, a true responder and a true non-responder, highlighting the model’s ability to explain its predictions on a per-sample basis. Both genomic and transcriptomic biomarkers have contributed to these predictions.

## 4. Discussion

Taken together, our data showed that AI models using multiple DNA- and RNA-seq-based biomarkers improved the prediction of anti-PD1 outcomes compared to the current gold standard, nsTMB. All trained ML models, using only genomic or transcriptomic biomarkers, as well as the multi-omic model, outperformed nsTMB on the training and the test set in terms of the ROC AUC, suggesting that incorporating additional features can provide a more accurate prediction of anti-PD1 responses if sufficient training data are available. However, the availability of homogeneous training data is still unsatisfactory, as even after combining multiple studies into a meta-cohort, the number of samples with both WES and RNA-seq remained limited. Therefore, we expect that larger training datasets will further improve the potential of AI models.

Although the cost of a multi-omics analysis (WES + RNA-seq) is higher than, e.g., a small gene panel, this approach provides not only a comprehensive view on tumor immunogenicity but also various treatment-relevant biomarkers such as fusion gene events, the overexpression of amplified oncogenes, the depletion of deleted tumor suppressor genes, as well as an evaluation of splicing defects. As sequencing costs continue to decline, the wider adoption of combined WES and RNA-seq may become more feasible in routine clinical practice, where even small improvements in prediction accuracy may have clinical benefits, particularly in the selection of patients for ICI.

As anti-PD1 treatment is frequently considered the last treatment option, patients frequently receive ICIs, regardless of the outcome of tumor genome diagnostics. As a result, the identification of discriminative features between responders (true positives) and non-responders (true negatives) is of particular interest to increase the explainability of the results, potentially leading to improved diagnostic interpretation. We achieved patient-specific explainability of the outcome by using SHAP values (Figure 5c). These provide clinicians with a detailed breakdown of the biomarkers that contributed most to the classification of a patient. Patients showing strong indicators of non-respondents could receive another type of therapy, such as *BRAF* inhibitors, or could be spared from the side effects of the treatment.

Surprisingly, the pure RNA model (without DNA-based features) achieved a better overall performance than the well-established gold standard nsTMB alone in both the test and training datasets, although it did not include nsTMB or any other TMB-related metric. This emphasizes the potential of RNA-based features in modeling ICI responses, mostly based on features reflecting immune cell infiltration (quantified as TCR composition) and interferon signatures. Integrating DNA- and RNA-based biomarkers into a multi-omics model resulted in higher overall performance, despite the substantially reduced training dataset for which both WES and RNA-seq were available. Feature selection for the multi-omics model indicated that RNA-derived biomarkers were mostly independent of DNA-derived biomarkers, contributing equally to the top four most influential features. Indeed, we did not detect strong correlations between the genomic and transcriptomic biomarkers in the training dataset (Figure S3). However, we observed correlations between RNA-seq-based biomarkers and tumor purity. Hence, models that include RNA biomarkers may underperform in low-purity samples.

The features preferentially selected by the DNA and RNA models were highly biologically relevant (Figure 3a,b). The DNA model favored, for example, in addition to nsTMB, the related metric fsIndel burden. Frameshift mutations are a rich source of highly immunogenic neoepitopes that are recognized by the immune system [42]. In addition, the DNA model benefitted strongly from information about genetic variants in response pathways and resistance mechanisms, with changes in response pathways (*CTNNB1* alterations, *JAK1*/*JAK2* alterations, and defects at chr6p21.3) being the most important feature. Alterations in *B2M* are the most influential feature representing mutated resistance mechanisms. *B2M* alterations can lead to a dysfunctional MHC complex. This mechanism is crucial for antigen presentation by cancer cells [43]. Notably, no biomarker related to the predicted neoantigens exceeded the feature permutation importance threshold of 0.0055. Therefore, we assume that nsTMB is a comprehensive proxy for most neoantigen-related biomarkers. In contrast, the RNA model favored immune-related features, such as TCR α chain entropy, IFNG-IMS ratio, and IGH chain entropy, with TCR α chain entropy being by far the most important biomarker. These features capture many aspects of the tumor immune microenvironment [44]. TCR α and IGH chain entropy reflect information regarding immune cell infiltration and diversity in tumors of both T cells and B cells. With IFNG-IMS, the RNA model also selected an important marker of immune balance because it combines transcriptomic signals of immune response and immunosuppression [45]. All features selected by the DNA and RNA model retained non-zero coefficients in the multi-omics model. However, the permutation feature importance scores differed for some features in the multi-omics models compared with those in the DNA and RNA models. For example, tumor purity had a moderate feature importance score in the DNA model but was almost zero in the multi-omics model. This can be attributed to the correlation between tumor purity and TCR α chain entropy (Figure S3). Tumors with low purity often have higher immune cell infiltration, leading to higher TCR α chain entropy.

The SHAP analysis of true responders and true non-responders in the multi-omics model confirmed the importance of the DNA and RNA features that were originally selected by feature permutation importance (Figure 5a–c). Remarkably, the SHAP methodology allowed us to directly determine the direction in which biomarkers influenced the predictions. For example, it became evident that high TCR α chain entropy or a high nsTMB and fsInDel burden contributed to positive predictions as responders. The SHAP analysis confirmed that patients with mutated response pathways were more likely to respond to immunotherapy, whereas patients with defects in *B2M* were less likely to be classified as responders. More importantly, the application of SHAP values demonstrated the ability of our model to explain the biomarker contribution on a single-sample basis, i.e., the classification of a specific patient. In Figure 6, we showed two representative SHAP examples of single-sample predictions from the multi-omics model, providing further evidence for the per-patient interpretability of our predictions. This is an important step towards the clinical usability of machine learning models because the selection of a patient for anti-PD1 therapy requires individual explainability and assessment of the classification.

Despite showing promising results, the overall improvements in treatment response prediction of the three models over nsTMB are still unsatisfactory. One reason could be the exclusion of non-NGS patient data in our models, such as data on the gut microbiome, comorbidities, age, alcohol consumption, or serum albumin. Some of these factors, such as the gut microbiome [46], or serum albumin level [47], have been shown to correlate with ICI outcomes in various cancer types. Some studies have used AI models to investigate the interplay between clinical non-NGS features and ICI [48]. However, we did not find sufficient data in available melanoma studies capturing a comprehensive selection of such biomarkers together with genomic and transcriptomic data in melanoma. Future studies should aim to collect a broader range of clinical non-NGS biomarkers, in combination with NGS data.

Another limitation of our study is that it combined data from different treatment centers into a meta-cohort. Although we applied several strategies to exclude batch effects (see Figure S1), we cannot rule them out. Moreover, we did not consider information regarding prior treatments or alternative treatment options for the patients, as it was generally not available. Interestingly, only 128 (28%) of the 449 WES samples in our meta-cohort had a *BRAF*:V600 alteration, while the expected prevalence of such a mutation is approximately 50% in cutaneous melanoma [49]. This discrepancy suggests that around half the tumors with a *BRAF* alteration may have been treated with *BRAF* inhibitors and not with ICIs, potentially introducing a selection bias. As a result, our findings may not be fully generalizable to the entire melanoma spectrum, particularly for patients harboring *BRAF* mutations (although 128 *BRAF*:V600 cases are a sufficient number to inform model training). Finally, consistent staging information was not available for all studies. As melanoma stage may influence treatment selection and response to anti-PD1 treatment, this limits the assessment of our models regarding specific stages.

The limited size of the meta-cohort, especially the number of samples with both WES and RNA-seq, was the main constraint. Therefore, using a larger cohort would likely improve model training and evaluation. It should also be noted that RECIST, as an outcome measure, is a commonly used but not universally robust metric [50]. Patients with stable disease may have survival benefits, whereas partial responders may progress to a later stage. However, response measures other than the RECIST are often unavailable for public studies. Collecting more comprehensive response data could be a starting point for future trials to address this issue. Despite these limitations, we demonstrated the general usability and interpretability of multi-omics LASSO models for ICI outcome prediction in cancer.

## 5. Conclusions

In this study, we combined genomic and transcriptomic biomarkers of a large, melanoma-specific meta-cohort to train a multi-omics LASSO model to predict the response to ICI. Multiple biomarkers derived from both whole-exome sequencing and RNA-seq were selected as influential features of the multi-omics model. They showed promising improvements in predicting anti-PD1 outcomes compared with nsTMB alone. The final feature selection revealed that biomarkers describing the tumor immune microenvironment should be considered together with tumor cell-intrinsic mutation-based metrics. By applying the SHAP technique, the model predictions in this study were easier to interpret and explainable on a per-patient basis, making them more applicable in clinical decision-making. Although our study demonstrated the general applicability and explainability of multi-omics LASSO models for ICI outcome prediction, the predictions are not yet fully satisfactory. The main limitations of our study were the limited size of our meta-cohort and limited homogeneity. Future research should therefore aim at collecting large homogeneous ICI cohorts analyzed by WES and RNA-seq for AI model training.

## Data Availability

The NGS data used for this study were compiled using eight different, publicly available melanoma datasets. These datasets can be obtained from SRA, from the gene expression omnibus, or from dbGaP (after data access request) under the following study accession numbers: Amato et al. [22]: PRJNA639866, GSE15996; Cristescu et al. [23]: phs001572.v1.p1; Gide et al. [24]: PRJEB23709; Hugo et al. [25]: SRP090294, SRP067938, GSE78220; Liu et al. [26]: phs000452.v3.p1; Pyke et al. [27]: phs002388.v1.p1; Riaz et al. [28]: SRP094781, GSE91061; Wolchok et al. [29]: SRP417444.

## Supplementary Materials

The following supporting information can be downloaded at https://www.mdpi.com/article/10.3390/cancers17050714/s1, Figure S1. Principal Component Analysis (PCA) of the expression of all genes and samples (in TPM). Each dot color represents one sub-cohort constituting our meta-cohort. We plotted the principal components with the highest explained variances. Left: PCA before batch correction. Batch effects were clearly visible. Right: PCA after batch correction using pyComBat. Batch effects were no longer visible. Figure S2. Distribution of the predicted response probabilities of the models in the training datasets. The probability density is different in every model, emphasizing the necessity of model-specific probability cutoff values. The vertical lines represent these thresholds, which were determined by maximizing Youden’s indices. Figure S3. Correlation heatmap of the biomarkers in the multi-omics model. We observed only weak correlations between the transcriptomic and genomic biomarkers. However, we can clearly recognize a cluster of transcriptomic biomarkers. We used Pearson’s ρ and applied hierarchical clustering to arrange all features into clusters. Only the data from the training set of the multi-omics model were used to generate this plot. Table S1. Resistance mechanisms.

## Appendix A

### Appendix A.1. DNA Biomarkers

#### Tumor Mutational Burden and Related Mutation Count Metrics

To calculate nsTMB, we considered only nonsynonymous variants that occurred in the overlapping target region of all studies. nsTMB was calculated as the number of nonsynonymous variants divided by the size of the target region in megabases. In addition to the classical nsTMB, we derived related burden metrics using different mutation types, normalized by the size of the common target region. We determined the different burdens using the following mutation types: (i) indels, (ii) frameshift indels, (iii) in-frame indels, (iv) splice variants, (v) missense variants, (vi) synonymous variants, and (vii) variants that cause changes in multiple amino acids (i.e., splice and frameshift). The frameshift mutation proportion, another TMB-related biomarker, was also calculated. It is defined as the proportion of frameshift mutations among all the mutation types. Its predictive potential for anti-PD1 therapy has recently been described in a different cancer entity [51]. In addition to these TMB-related metrics, we included the ratio of all nonsynonymous mutations to all synonymous mutations, known as the dN/dS ratio [52].

### Appendix A.2. Neoantigen and Neoepitope Analysis

The binding affinities to major histocompatibility complex (MHC) class I molecules were predicted using pVACseq 4.0.1 for all nonsynonymous somatic variants that lie in the common target region [53]. This pipeline was executed for all possible peptides with lengths of 8, 9, and 10, reflecting the most common MHC class I peptide-binding lengths [54]. We executed pVACseq using the neopeptide-binding prediction algorithms MHCflurry, MHCnuggetsI, NNalign, NetMHC, NetMHCpan, and NetMHCpanEL. We used local instances of the IEDB MHC class I-binding prediction tools 3.1.2 and MHCflurry 2.0.6 for the pVACseq predictions. We then filtered neoantigen candidates using a binding affinity cutoff of IC50 < 50 nM. After filtering, neoantigen counts were normalized to the size of the target in megabases, resulting in a TMB-like biomarker called neoantigen burden. To account for neoantigen quality, we used the binding affinity of the biomarker with the highest binding affinity as a feature. Another neoantigen metric, the differential agretopicity index (DAI), describes the difference between the predicted binding affinities of wild-type and mutated alleles [55]. Values < 0 indicate a higher binding affinity for the mutated peptide than for the wild-type peptide. We calculated the mean, median, upper decile, and maximum values of all DAIs as biomarkers, reflecting both neoantigen quantity and quality [56].

We passed any neoepitope candidate with a median binding affinity cutoff of IC50 < 500 nM to the neoantigen annotation tool NeoFox 1.1.0 to obtain further neoantigen features [57]. Three metrics predicted by NeoFox were integrated into our model. They describe various properties of the neoepitope candidates:

- HEX alignment score: This score describes the similarity of tumor peptides to known viral-derived peptides [58]. High scores reflect a high similarity of the neopeptide with viral pathogens and could be more likely recognized by the immune system. We used the maximum HEX score in the sample as a biomarker.
- Recognition potential: A fitness model that uses binding affinities and sequence similarity of neoantigens to known pathogens of human infectious diseases to estimate the likelihood of a neoantigen interacting with the immune system [59]. Its maximum value was used as a biomarker for our model.
- Dissimilarity score: This score compares the mutated peptide to the non-mutated self-proteome [60]. Neopeptides with higher dissimilarity scores are more likely to be recognized by the immune system. Therefore, we used the maximum dissimilarity score as the biomarker.

### Appendix A.3. Resistance Mechanisms

Resistance mechanisms are those by which cancer cells evade cancer treatment. In ICI, these mechanisms are typical ways in which tumors can escape immune responses. Following a literature review, we included several such biomarkers in our model. Resistance mechanisms are usually described as deleterious mutations in genes of certain pathways.

Frequent mechanisms include defects in the HLA antigen presentation pathway, which prevents T cells from recognizing cancer cells [61]. Most genes that form MHC class I molecules reside at chr6p21.3. Thus, we defined a biomarker that describes deleterious events at this locus: any deletion or loss of heterozygosity (LOH) event at chr6:29941259-33314212. This region contains all HLA class I genes and genes necessary for antigen processing, such as the transporter proteins *TAP1*/*TAP2* and *TAPBP*. Due to the highly polymorphic nature of this locus, we did not include potentially deleterious SNVs and small indels [62]. However, we included deleterious SNVs and indels in *B2M* as independent biomarkers. We assumed a dysfunctional *B2M* protein if we detected LOH events, heterozygous deletions, mutations with a high VEP effect annotation, or variants with an annotation of COSMIC CMC tiers 1, 2, and 3. In addition to these two HLA-related mechanisms, a complete list of all the resistance mechanisms integrated into our models can be found in Table A1.

Some of the mechanisms affected only a small proportion of our training dataset. Thus, we merged the mechanisms affecting less than 20% of the training dataset into one biomarker. This resulted in the union of defects at the HLA locus, CTNNB1 alterations, and JAK/JAK2 alterations as one biomarker. An analysis of odds ratio revealed that these biomarkers were more enriched in responders than in non-responders. That is why we call the resulting merged biomarker a “response pathway”. Further details are provided in Supplementary Table S1.

**Table A1:** Resistance mechanisms. Each mechanism represents a binary biomarker (true or false). If one of the described alterations was detected, the biomarker was set as true. If none of the described alterations were found, the corresponding biomarker was set as false. Mechanisms marked by an asterisk (*) affected less than 20% of the training cohort and were merged into one biomarker.

Mechanism	Description	Sources
Defects at the HLA locus *	Any deletion or LOH event that overlaps chr6p21.3, chr6:29941259-33314212. This locus contains the majority of HLA class I-related genes.	[61]
Defects in *B2M*	*B2M* is an integral part of both MHC class I and II complexes. Dysfunctional *B2M* proteins lead to the formation of dysfunctional MHC complexes. However, the loss of both functional alleles is uncommon since such cancer clones are eradicated by NK cells. Hence, we counted heterozygous deletions; LOH events; mutations with a high VEP effect; or COSMIC tiers 1, 2, and 3 as defects in *B2M*.	[63]
*JAK1*/*JAK2* alteration *	Homozygous deletions; VEP-high; or CMC tier 1,2, and 3 somatic variants of *JAK1* and *JAK2*. Other genes of the JAK-STAT pathway were not included because *JAK3* is not expressed in solid tissue and alterations in most STAT genes are difficult to interpret because STATs are, at the same time, both TSG and oncogenes.	[64]
*CTNNB1* pathway alteration *	*CTNNB1* is an oncogene. Its pathway includes the negative regulators *APC*, *AXIN1*, and *HNF1A*, which are present in our intersecting target region. Thus, we counted the COSMIC tier 1, 2, and 3 mutations of *CTNNB1*. For its negative regulators, which act as TSG, we considered any somatic mutation with a high VEP effect or COSMIC tiers 1, 2, and 3 as well as homozygous deletions.	[65]
*TP53* alteration	*TP53* is frequently mutated in many tumors. Defects in this TSG have manifold effects on cancer cells, including immunosuppression and evasion. Thus, we considered any deletion; LOH; mutation with a high VEP effect; or COSMIC tiers 1, 2, and 3 in *TP53*.	[66]
*PTEN* alteration	Deleterious events in the TSG *PTEN* were described as a resistance mechanism previously. Thus, we considered any deletion; LOH; mutations with a high VEP effect; or COSMIC tiers 1, 2, and 3 in *PTEN* as deleterious.	[67,68]
*STK11* alteration	*STK11* is a tumor suppressor gene. It was predictive of anti-PD1 resistance in a study on another cancer entity. Hence, we counted any deletions; mutations with a high VEP effect; or COSMIC tiers 1, 2, and 3 in *STK11*.	[69]
*KRAS* alteration	*KRAS* is an oncogene. The predictive potential of *KRAS* mutations in anti-PD1 treatment has been previously described in another cancer entity. Thus, we considered any amplification ≥ 4 and mutations with COSMIC tiers 1, 2, and 3 in *KRAS* deleterious.	[70]
*MDM2* alteration	*MDM2* is an oncogene. Activating mutations led to the hyperprogression of melanoma in a previous study. Thus, we counted any amplification ≥ 4 and COSMIC tiers 1, 2, and 3 in *MDM2* as resistance mechanisms.	[71]
*MDM4* alteration	*MDM4* is an oncogene. Activating mutations led to the hyperprogression of melanoma in a previous study. Thus, we counted any amplification ≥ 4 and COSMIC tiers 1, 2, and 3 in *MDM4* as resistance mechanisms.	[71]
*EGFR* alteration	*EGFR* is an oncogene. Activating mutations led to the hyperprogression of melanoma in a previous study. Thus, we counted any amplification ≥ 4 and COSMIC tiers 1, 2, and 3 in *EGFR* as resistance mechanisms.	[71]

### Appendix A.4. HLA-Related Biomarkers (Germline)

Carriers of homozygous HLA alleles are thought to be at a considerable disadvantage because their MHC complexes bind to a significantly smaller neopeptide repertoire than that of heterozygous individuals. Indeed, some studies have shown that patients homozygous for *HLA-B* and *HLA-C* are less likely to respond to ICI [72]. Therefore, we included both *HLA-B* and *HLA-C* homozygosity as binary biomarkers.

HLA evolutionary divergence (HED) is a measure of the evolutionary protein distance between HLA alleles in an individual [73]. It has been described as a predictive biomarker for anti-PD1 therapy outcomes in patients with melanoma [74]. In this study, we used HED, quantified as the Grantham distance between both alleles. We included the mean HED of all three classical HLA loci (*HLA-A*, *HLA-B*, and *HLA-C*) as germline biomarkers in the model. We used an in-house tool to calculate HED, available at GitHub https://github.com/axelgschwind/hlaDivergence (commit 37bd5fc, accessed on 17 April 2023).

HLA alleles can be grouped into several supertypes based on their similar physicochemical properties. The three *HLA-B* supertypes HLA-B27, HLA-B44, and HLA-B62 have been described as predictors of ICI outcomes in melanoma and are thus included as HLA-related binary biomarkers [72,75].

### Appendix A.5. Tumor Purity and Heterogeneity

Tumor purity was defined as the percentage of cancer cells in a tumor sample. It has been described as a predictor of ICI outcomes in various types of cancer [76]. In this study, we defined tumor purity as the highest value of tumor clonality determined by ClinCNV. No CNVs were detected in very few samples. In these cases, we used the median VAF of the 15 SNVs with the highest VAF as an estimate of purity.

### Appendix A.6. CNV Burden

We defined the CNV burden as the proportion of the genome that carries a CNV to the total size of the genome. We only considered CNVs that passed quality control and autosomal CNVs. The deletion burden is a related biomarker and is defined as the proportion of the genome that carries a heterozygous or homozygous deletion.

### Appendix A.7. RNA Biomarkers

#### TCR and BCR Repertoires

We used TRUST4 to reconstruct TCR and BCR clones from RNA-seq data [77]. We obtained clones of the TCR α, TCR β, and BCR IGH chains. Clones with fewer than two reads were excluded from analysis. We then calculated the Shannon entropy based on the counts of distinct clones for each chain in each sample. This metric integrates both richness (number of distinct clones) and evenness (diversity index and distribution of clone counts) into a single, continuous biomarker.

### Appendix A.8. Immune Cell Infiltration

We estimated the composition of immune cell types from RNA-seq expression data using the deconvolution algorithm quanTIseq, as implemented by the wrapper pipeline immunedeconv 2.1.0 [78,79]. Using this approach, we can directly interpret the results as cell proportions in the RNA sample. In our work, we used the sum of the original quanTIseq cell types “B cell”, “T cell CD4+ (non-regulatory)”, “T cell CD8+”, “T cell regulatory (Tregs)”, and “NK cell” as a rough estimate for the lymphocyte infiltration of the sample.

### Appendix A.9. IFNG-IMS Ratio

We used the ratio of two immune-related gene expression signatures as a biomarker. The impact of this ratio has previously been described as a predictor of ICI outcomes in melanoma [80]. The first signature contained interferon-γ-related genes. The second signature described patterns of immunosuppression, originally obtained by comparing ICI responders with non-responders. Using the ratio of the signatures, both inflammatory and immunosuppressive elements were combined to predict the ICI outcomes more precisely. Following [80,81], the IFNG signature was calculated as the arithmetic mean of the read counts of *IFNG*, *STAT1*, *IDO1*, *CXCL9*, *CXCL10,* and *HLA-DRA*. The immunosuppression signature was defined as the mean expression values of *ADAM12*, *AXL*, *BCAT1*, *CCL13*, *CCL2*, *CCL8*, *CD163*, *COL6A3*, *FAP*, *IL10*, *INHBA*, *ISG15*, *OLFML2B*, *PDGFRB*, *SIGLEC1*, *STC1*, *TWIST2*, and *VCAN* [80].

### Appendix A.10. Gene Expression Profiles

Loss of HLA class I expression is a well-described mechanism of immune evasion and affects anti-PD1 treatment in melanoma [82]. Thus, we calculated the mean gene expression values (in TPM) of the three classical HLA class I loci, *HLA-A*, *HLA-B*, and *HLA-C,* as GEP biomarkers. *B2M* is also crucial for the formation of MHC complexes, and loss of its expression has been associated with anti-PD1 outcomes in a recent study of another cancer entity [83]. For our model, we used the gene expression of *B2M*, which was quantified as TPM. Additionally, the expression of the *PD1* ligand *PDL1* in terms of TPM was utilized as another GEP biomarker. It was included because it serves as an “official” indication for anti-PD1 treatment in other cancer entities.
