# Supplementary material for: AI Model for Predicting Anti-PD1 Response in Melanoma Using Multi-Omics Biomarkers
Source: Cancers (Basel). 2025 Feb 20;17(5):714. doi: 10.3390/cancers17050714 (PMC11899402; doi:10.3390/cancers17050714)
Supplement: Supplementary file 1 [file cancers-17-00714-s001.zip › cancers-3448291-supplementary-Figures.pdf]

Supplementary Figures

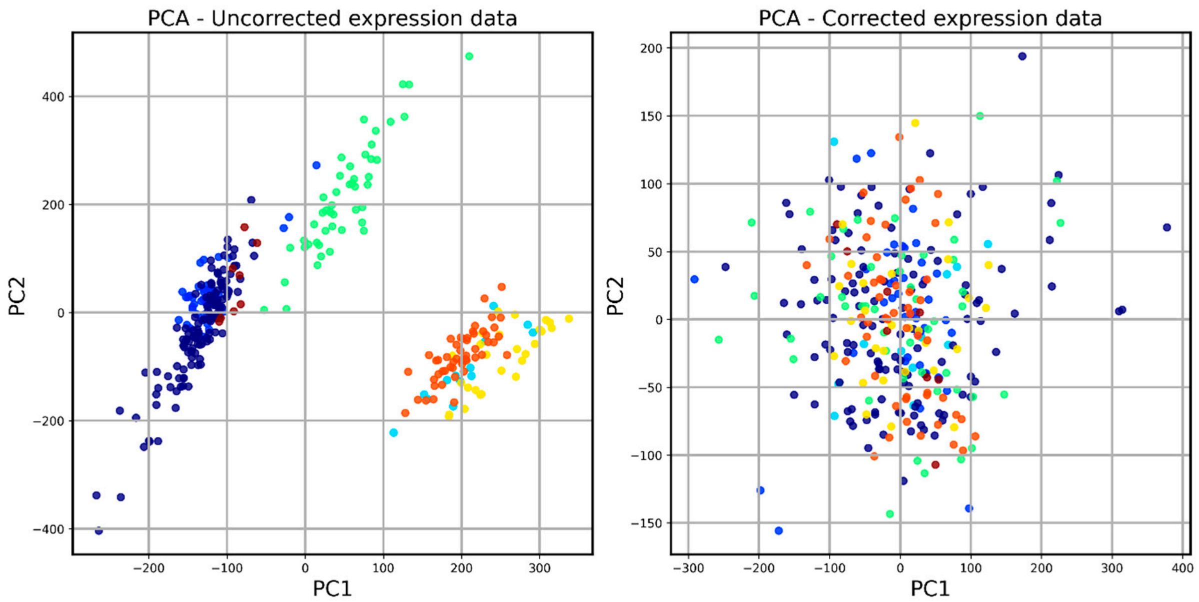

Figure S1. Principal Component Analysis (PCA) of the expression of all genes and samples (in TPM). Each dot color represents one sub-cohort constituting our meta-cohort. We plotted the principal components with the highest explained variances. Left: PCA before batch correction. Batch effects were clearly visible. Right: PCA after batch correction using pyComBat. Batch effects were no longer visible.

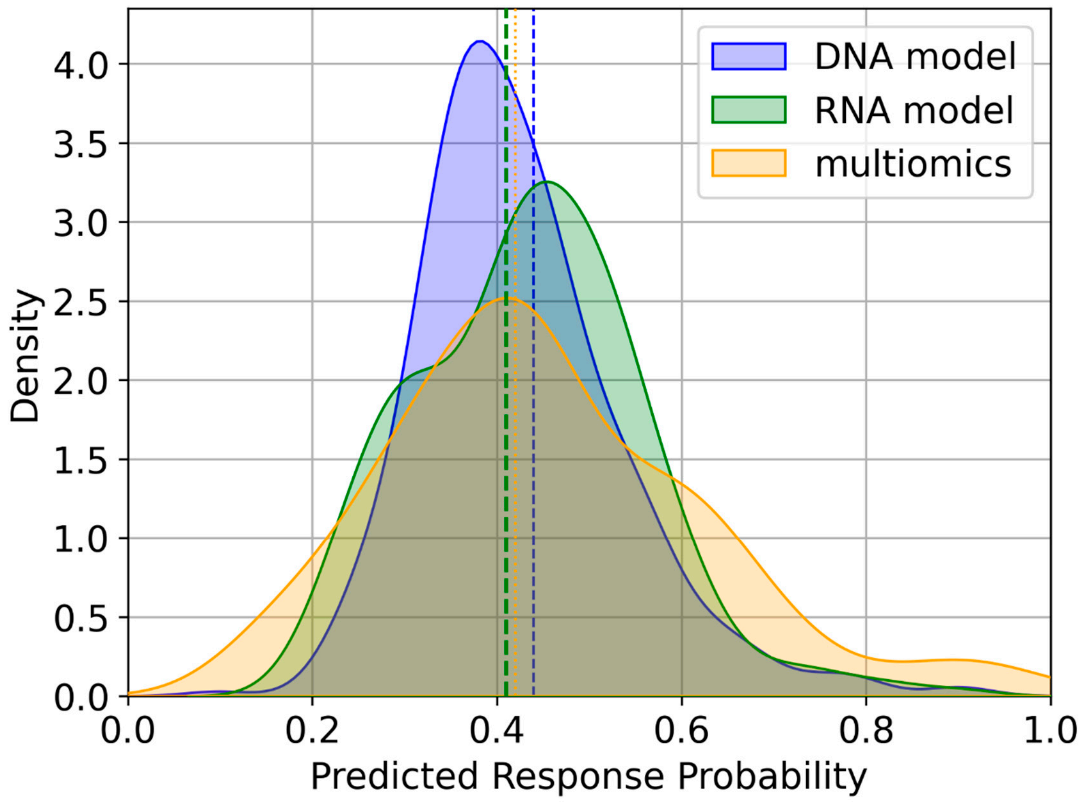

Figure S2. Distribution of the predicted response probabilities of the models in the training datasets. The probability density is different in every model, emphasizing the necessity of model-specific probability cutoff values. The vertical lines represent these thresholds, which were determined by maximizing Youden's indices.

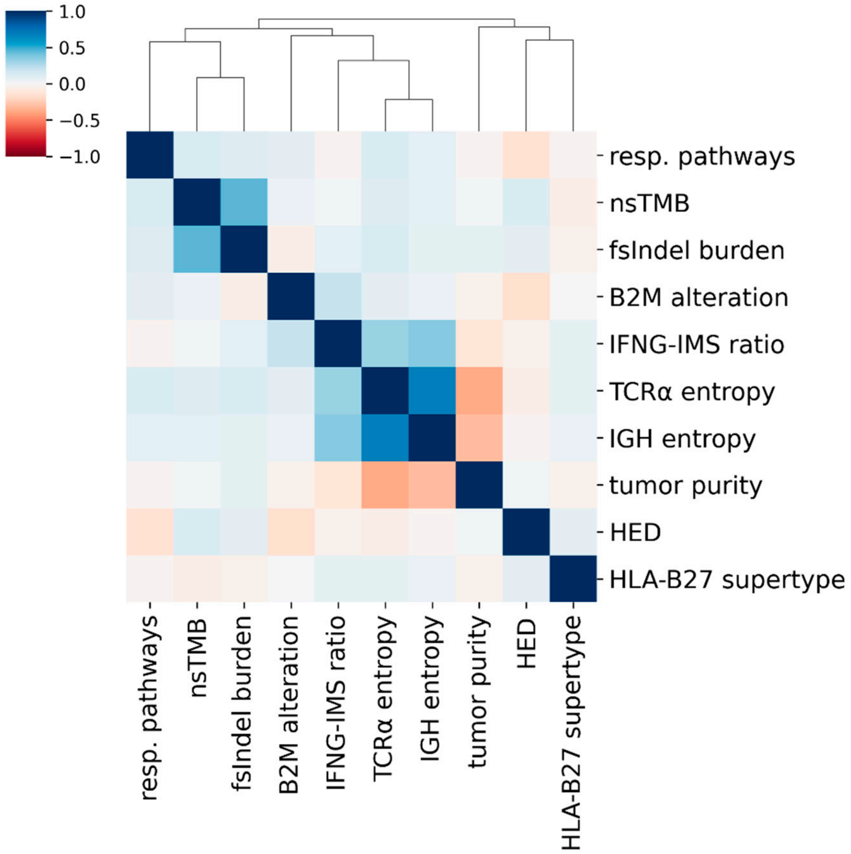

Figure S3. Correlation heatmap of the biomarkers in the multi-omics model. We observed only weak correlations between the transcriptomic and genomic biomarkers. However, we can clearly recognize a cluster of transcriptomic biomarkers. We used Pearson's ρ and applied hierarchical clustering to arrange all features into clusters. Only the data from the training set of the multi-omics model were used to generate this plot.
